# Identification of Key Pathways and Candidate Genes Controlling Organ Size Through Transcriptome and Weighted Gene Co-Expression Network Analyses in Navel Orange Plants (*Citrus sinensis*)

**DOI:** 10.3390/genes16030259

**Published:** 2025-02-23

**Authors:** Yi Lin, Jinghao Zheng, Qinyu Wan, Zhenmin Chen, Qiong Chen, Shubei Wan, Jianmei Chen

**Affiliations:** National Navel Orange Engineering Research Center, College of Life Sciences, Gannan Normal University, Ganzhou 341000, China; 1221016007@gnnu.edu.cn (Y.L.); zjh2610080410@163.com (J.Z.); 18270296978@163.com (Q.W.); chen002529@163.com (Z.C.); qiong0552@163.com (Q.C.); wanshubei@gnnu.edu.cn (S.W.)

**Keywords:** *Citrus*, organ development, gene expression, hub gene

## Abstract

**Background/Objectives:** Organ size is a critical target trait in fruit-tree breeding programs, as it significantly impacts the economic value of plants by influencing their biomass, yield, and quality. Understanding the molecular mechanisms underlying organ size in citrus is essential for breeding new cultivars with superior fruit quality. **Methods:** In this study, we investigated the regulatory network involved in organ size using the *Citrus sinensis* ‘Newhall’ navel orange variety and its large-organ mutant, ‘M25’. **Results:** Ploidy analysis indicated that the organ enlargement observed in ‘M25’ was not attributable to changes in chromosome ploidy. Furthermore, RNA sequencing of tender leaves and young fruits from both ‘M25’ and ‘Newhall’ oranges identified 1817 and 1605 differentially expressed genes (DEGs), respectively. Functional enrichment analysis revealed that these DEGs were enriched in pathways associated with organ size regulation, including those related to cell division, DNA replication, protein biosynthesis, plant hormone signal transduction, and cell wall metabolism. Weighted gene co-expression network analysis identified the grey 60 and orange modules as the key modules influencing organ enlargement; from these modules, we identified 51 and 35 hub genes, respectively. Combined homologous function annotation and expression analysis identified four transcription-factor-encoding hub genes (*Cs_ont_6g005380*, *Cs_ont_8g025330*, *Cs_ont_9g019400*, and *Cs_ont_9g008010*) as candidate genes potentially related to organ size. **Conclusions:** Among these, *Cs_ont_8g025330* (*CsMYB73*) was inferred to be the key gene influencing organ size through auxin and cytokinin regulation. These findings lay the foundation for further investigations of the regulatory mechanism of organ size in navel orange varieties.

## 1. Introduction

Organ size is a crucial aspect of plant morphology and significantly impacts fruit quality. *Citrus* varieties are among the most widely cultivated horticultural fruits due to their high economic value [1,2]. The size and shape of citrus plant leaves and fruits, which are typically assessed in terms of biomass, yield, and quality, directly influence the economic value of citrus varieties [3,4]. Therefore, research on the organ size of citrus fruits is vital in the fields of plant developmental biology and genetic improvement.

Many studies have investigated plant organ size, particularly in *Arabidopsis thaliana* (L.) Heynh. and the tomatillo *Physalis philadelphica* Lam. The size of a plant organ is determined by both the number and size of cells constituting the organ, which are correlated with cell division and cell expansion rates, respectively [5,6]. Several cell division or expansion genes are associated with plant organ size [7,8]. In *Arabidopsis*, cyclin D3 (CYCD3) genes regulate the cell numbers of developing lateral organs and the relative contributions of cell production and expansion to overall organ growth [7]. In *Medicago truncatula* Gaertn., the gene *MINI ORGAN1* (*MIO1*) encodes the SMALL LEAF AND BUSHY 1 (SLB1) F-box protein, which affects organ size as part of the SKP1/Cullin/F-box (SCF) E3 ubiquitin ligase complex, to target the primary cell division repressor BIG SEEDS1 (BS1) for degradation [8]. Recently, significant progress has been made in the study of leaf size regulation in Arabidopsis thaliana. Research has demonstrated that various growth regulators, including a ubiquitin-activated protease (DA1), the TIFY protein family (PPD), cytochrome P450 proteins (KLU), growth regulating factors (GRFs), switch/sucrose non-fermenting (SWI/SNF) complexes, and DELLA proteins (aspartic acid–glutamic acid–leucine–leucine–alanine), play crucial roles in this process [9]. In contrast, studies on ovary and fruit development have primarily focused on tomato (*Solanum lycopersicum*). Silencing the GAI-RGA- and SCR proteins 2 (GRAS2) gene in tomato results in reduced activity of gibberellin biosynthesis and signal transduction pathways, leading to insufficient levels of active gibberellins during early ovarian development. Consequently, this reduction affects fruit size by regulating ovary growth and cell size [10].

To date, several regulatory mechanisms governing plant organ size have been identified, including hormones, transcription factors, and non-coding RNAs. Phytohormones play crucial roles in plant development and have been shown to participate in the regulation of organ size. For instance, auxins, gibberellins, brassinosteroids, and cytokinins have been reported to be involved in this regulatory process [11]. In apple trees, high concentrations of auxin can significantly inhibit the expression of the *MdAux/IAA2* gene, resulting in enlarged fruit cell morphology that increases fruit size [12]. Gibberellins promote cell growth and root development. In tomato plants, the knockout of Gibberellin 20-oxidase (GA20ox), a key rate-limiting enzyme in gibberellin biosynthesis, resulted in a dwarf phenotype, whereas its overexpression produced a taller phenotype [13]. Brassinosteroids, which are steroid hormones essential for plant growth and development, play a significant role in this process. In *Arabidopsis*, the overexpression of Brassinosteroid-insensitive-1 (BRI1) in association with receptor kinase 1 (BAK1) enhances brassinosteroid signaling, influencing various aspects of plant growth, such as producing narrower rosette leaves, longer petioles, and a larger overall architecture [14]. Cytokinins play a key role in regulating cell division and expansion during plant growth. In kiwi plants, *ISOPENTENYLTRANSFERASE* (*IPT*) is a cytokinin biosynthetic gene that increases fruit growth by controlling the levels of endogenous cytokinins [15]. Thus, organ development is a complex process regulated by plant hormones. Transcription factors also play important roles in regulating organ size. AINTEGUMENTA (ANT) transcription factors belong to the AP2/ERF transcriptional regulatory family and are important regulators influencing plant organ growth. ANT overexpression in plants leads to larger leaves and flowers, whereas loss-of-function *ant* mutants produce smaller leaves [16]. A few non-coding RNAs have been identified as regulating organ size. In apple trees, *microRNA172* negatively regulates fruit size by inhibiting *AP2* gene expression [17]. *MiR159* positively regulates the size of rice plant organs, including stems, leaves, and fruit, by modulating the expression of the miRNA-targeted genes *OsGAMYB* and *GAMYB-LIKE 1* (*OsGAMYBL1*) [18]. In *Rosa hybrida*, miR159 controls cytokinin catabolism in the form of the MYB73/TPL/HDA19–miR159–CKX6 module [19].

However, few regulatory mechanisms of citrus organ size have been reported to date. The *R2R3-MYB* gene *CsMYB77* negatively regulates fruit size by modulating auxin homeostasis through positive regulation of PIN-FORMED PROTEIN5 (PIN5) [20]. Transcriptome analysis showed that 17 genes, including two *Fruit Weight* (*FW*) genes and three *Expansin* (*EXP*) genes, were differentially expressed among a set of somatic varieties with fruit size differences [21]. In *Citrus*, the *LATERAL ORGAN BOUNDARY 1* (*CsLOB1*) gene was found to offer resistance to Citrus canker disease; overexpression of *CsLOB1* leads to pectin depolymerization and enhances expansin accumulation, resulting in dwarf phenotypes characterized by smaller, thicker leaves [22]. However, most such studies have examined only fruit size, and the molecular mechanisms underlying organ size modulation in citrus varieties remain poorly understood.

Newhall is a seedless citrus fruit cultivar widely cultivated in Ganzhou, located in the south of Jiangxi Province, China. A spontaneous large organ-size mutant of the Newhall navel orange (*Citrus*) was discovered in Ganzhou and designated as ’M25’. Compared to the Newhall navel orange, ’M25’ exhibits enlarged organ sizes, including increased dimensions of the stem, leaf, flower, and bud. This group of accessions, referred to as Newhall-M25, is particularly suitable for investigating the mechanisms that regulate organ size. RNA sequencing (RNAseq) and weighted gene co-expression network analysis (WGCNA) are appropriate approaches for exploring the regulatory mechanisms of organ size in these two citrus accessions [23,24]. In this study, we determined genome-wide gene transcription levels through RNAseq analysis of leaf and fruit samples to investigate the causes of the enlarged organs in the ’M25’ variety. Additionally, we employed WGCNA and quantitative real-time polymerase chain reaction (RT-qPCR) to identify hub genes potentially regulating organ size. Our findings elucidate the mechanisms underlying organ size in citrus varieties and provide fundamental data for future studies on plant organ development.

## 2. Materials and Methods

### 2.1. Plant Materials

The navel orange bud mutation M25, exhibiting enlarged organ size, was derived from a Newhall navel (NHE) orange at Ganzhou, Jiangxi Province, China. Ganzhou is located on the southern edge of the middle subtropical zone, with a subtropical hilly and humid monsoon climate. In this study, all materials were grown in the fields of the experimental nursery at Gannan Normal University. The cultivation and management were the same as local field cultivation conditions. Cultivation conditions for M25 plants were the same as those for the Newhall navel orange plants. The samples were picked and immediately placed in liquid nitrogen and kept at −80 °C until use.

### 2.2. Microscopic Analysis

The mature leaves of the branches of NHE and M25, which have ten aged trees, were selected for microscopic observations, and the two varieties had three leaves each. Samples were fixed in Carnoy’s solution (75% absolute ethanol and 25% acetic acid) at 4 °C for 6–12 h, then dehydrated through a graded ethanol series, cleared in dimethylbenzene, and embedded in paraffin [25]. Transverse sections (8 μm) were cut using a microtome (Leica RM2016, Leica, Wetzlar, Germany) and stained in toluidine blue. Bright-field photographs were taken using the Leica application suite. Cell size was measured using ImageJ/Fiji software (imagej.nih.gov). Data were analyzed for statistical significance using Student’s *t*-test.

### 2.3. Flow Cytometry

Polyploids sometimes have larger organs than their diploid counterparts, including larger and/or thicker leaves, flowers, or fruits [26]. The young leaf of the NHE and M25 mother trees was selected for ploidy variation detection, with three leaves from each of the two varieties. Samples were placed in a plastic culture dish with 500 μL Nuclei Extraction buffer (CyStain UV Precise P Kit, Sysmex, Beijing, China), chopped with a sharp blade, and then filtered through a 50 μm Celltrics filter after 60 s. Followed by the addition of 2000 μL of DAPI (4’,6-diamidino-2-phenylindole) staining buffer for 2 min in dark conditions. Nuclei suspension was analyzed by CyFlow Space Flow Cytometer (Sysmex Partec, Muenster, Germany) and the corresponding FloMax 2.1× software.

### 2.4. RNA Preparation

Total RNA was extracted from tissues with the FastPure Universal Plant Total RNA Isolation Kit (Vazyme, NanJing, China). RNA quality and concentration were determined by electrophoresis and spectrophotometry (Nanodrop ND-2000, Thermo Scientific, Waltham, MA, USA). The first-strand complementary DNA was synthesized using a reverse transcription kit HiScript III RT SuperMix for qRT-PCR (Vazyme, NanJing, China).

### 2.5. RNA-Seq Analysis

Transcriptome analyses of M25 were performed by RNA sequencing, with plants of Newhall navel orange serving as the control. Tender fruit at 10 days after the flowers (DAF) stage and young leaves were sampled for RNA-Seq analysis. The total RNAs were extracted. The library construction and sequencing were using the Illumina Hiseq (Illumina, San Diego, CA, USA). Raw data were preprocessed using the fastp (0.20.0) tool (http://github.com/openGene/fastp) (accessed on 19 February 2025). General statistics of sequencing data are listed in Supplementary Table S1. The clean reads were mapped to the *Citrus sinensis* *v*3.0 reference genome (http://citrus.hzau.edu.cn) (accessed on 19 February 2025) using HISAT2 (v2.1.0) with the default parameters (Supplementary Table S1) [27]. Differentially expressed genes (DEGs) (|log2 (Fold change)| ≥ 1, *p*-value < 0.05) were performed using DESeq2 (v1.26.0) [28]. The experiments used three biological replicates. Functional annotation of the DEGs was then conducted by searching against the SwissProt database by Gene Ontology (GO) annotation and the Eudicots plant database in the Kyoto Encyclopedia of Genes and Genomes (KEGG) database by using Basic Local Alignment Search Tool (BLAST) v2.10.1 [29]. The raw data of RNA-seq were uploaded to the National Biotechnology Information Center (NCBI) SRA database with the primary accession code PRJNA1173850.

### 2.6. WGCNA Analysis

The transcriptome data of these 12 samples contained fragments per kilobase of exon model per million mapped fragments (FPKM) data of all genes that were selected for gene co-expression network analysis of *C. sinensis*. We use the R package (https://cran.r-project.org/package=fragility) (accessed on 19 February 2025) WGCNA v1.6.1 for co-expression-network analysis as described previously [30,31]. A proper β value was selected to increase the matrix similarity and establish a co-expression network. After the network was generated, the correlation between modules and mutant traits was calculated. Then, referring to the method of Panahi et al. and Cui et al., the key module may be related to organ size in *C. sinensis,* which was identified based on the thresholds |R^2^| > 0.8 and *p* < 0.05 [32,33]. Based on the intersecting network, to screen out genes with high correlations, we set thresholds (MM (module feature) >0.8 and GS (gene saliency) >0.8) for the hub genes [33].

### 2.7. Quantitative Real-Time PCR

Quantitative real-time PCR (RT-qPCR) was performed on a LightCycler^®^ 96 Instrument Real-Time PCR Detection System (Roche Inc., Branchburg, NJ, USA) with ChamQ Universal SYBR qPCR Master Mix (Vazyme, Nanjing, China) using the gene-specific primers listed in Supplementary Table S1. The RNA used for qRT-PCR was extracted independently of that used in RNA sequencing, and each sample had three biological replicates. The calculation method of the relative expression of the gene is based on the method of Hellemans et al. [34]. Three biological replicates were performed for each treatment. Data were analyzed for statistical significance using Student’s *t*-test.

## 3. Results

### 3.1. Performance of the Navel Orange Mutant M25

**Figure 1:**
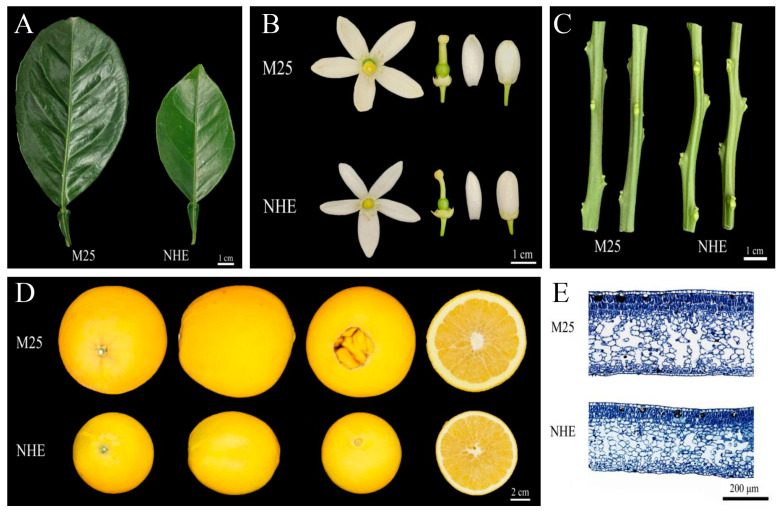
Performance of mutant M25 and Newhall navel orange. (**A**–**D**) were mature leaves (**A**), flowers (**B**), stems (**C**), and fruits (**D**) of M25 and Newhall navel orange, respectively. (**E**) Transverse sections of the leaf blade of the mature leaves in M25 and Newhall navel orange. The magnification of the microscope used to observe the samples was 100 times.

Organs were significantly larger in ‘M25’ naval orange plants than in ‘Newhall’ plants (Figure 1A–D). ‘M25’ plants had larger, darker, thicker, and more uneven leaves than ‘Newhall’ plants, which had flat leaves (Figure 1A). To assess the effects of these traits on photosynthetic performance in the ‘M25’ mutant, we examined the chlorophyll (Chl) contents of leaves of both varieties. Compared to those of ‘Newhall’, ‘M25’ leaves had significantly higher chlorophyll a (Chl a), chlorophyll b (Chl b), total Chl contents, and Chl a/b ratios (Supplementary Table S2). These findings indicate improved photosynthetic performance in the ‘M25’ mutant. ‘M25’ flowers had a thicker palea, larger ovary, wider petals, and larger petal area than ‘Newhall’ flowers (Figure 1B). The stem culm diameter was larger in the ‘M25’ mutants than in the ‘Newhall’ plants during the same growth phase (Figure 1C, Supplementary Figure S1A), and their fruits were larger and heavier (Figure 1D, Supplementary Figure S1B–C). The ‘M25’ and ‘Newhall’ plants exhibited significant size differences throughout the fruit growth stage, even during ovary development.

To uncover the cellular basis of this mutant phenotype, we examined the anatomical structure in leaf transverse sections using paraffin sectioning (Figure 1E). The sizes of the spongy and palisade tissue cells, as well as the intercellular spaces between spongy mesophyll cells, were larger in the mutant compared to those in the NHE leaves (Figure 1E; Supplementary Figure S2). The increased cell volume contributes to the larger organ size.

### 3.2. Ploidy Analysis

**Figure 2:**
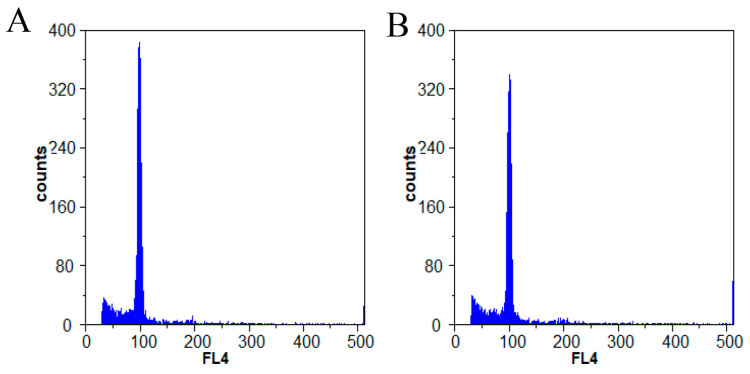
Ploidy levels of M25 (**A**) and Newhall (**B**) navel orange were monitored by flow cytometry. The ordinate indicates the number of cells, and the ordinate indicates the relative intensity of the fluorescence signal.

To determine whether the differences in organ size between the ‘M25’ and ‘Newhall’ navel orange varieties could be attributed to variations in ploidy, we conducted flow cytometry analysis to assess the ploidy levels of the ‘M25’ mutant. The ‘Newhall’ navel orange is a diploid variety. Our analysis revealed no differences in peak fluorescent signal intensity between the two varieties under the same conditions (Figure 2), suggesting that both varieties share the same ploidy level and are diploid. Consequently, the development of larger organs in ’M25’ cannot be attributed to changes in ploidy.

### 3.3. RNA-Seq Analysis

**Figure 3:**
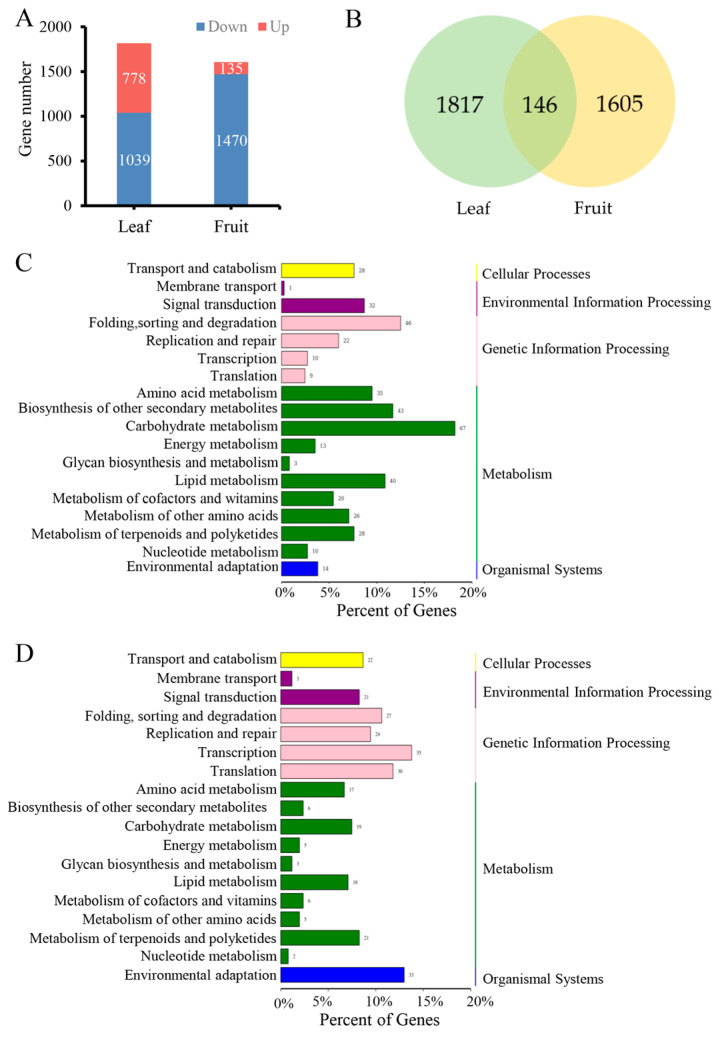
Identification and pathway enrichment analysis of DEGs related to organ size. (**A**) Number of DEGs; (**B**) Venn diagram analysis showing the number of common and unique DEGs identified in tender leaves and young fruits; (**C**,**D**) KEGG enrichment pathway (level 1 and 2) annotated classification results of all differentially expressed genes in M25 and Newhall navel orange in tender leaves (**C**) young fruits (**D**), respectively.

We performed RNA-seq analysis of tender leaves and young fruits in both navel orange varieties ‘Newhall’ and ‘M25’. ‘Newhall’ was used as a control. We identified 1817 DEGs in tender leaves (778 upregulated, 1039 downregulated), and 1605 DEGs in young fruits (135 upregulated, 1470 downregulated) (Figure 3A; Supplementary Figure S3A,B; Supplementary Table S4). Among these DEGs, 146 were differentially expressed in both tissues (Figure 3B). All DEGs were annotated using Basic Local Alignment Search Tool (BLAST) searches of the Kyoto Encyclopedia of Genes and Genomes (KEGG) and Gene Ontology (GO) databases.

KEGG pathway analysis was performed to identify metabolic or genetic information processing pathways enriched in all DEGs (Figure 3C,D). The results show that the DEGs were enriched in the genetic information processing, metabolism, cellular process, organismal system, and environmental information processing pathways. We further identified the 20 KEGG pathways that showed the greatest enrichment in both tissue types (Supplementary Figure S4A,B).

In leaves, we detected 36, 10, 11, and 11 DEGs enriched in the endoplasmic reticulum protein processing (*q* = 1.437 × 10^−3^), phenylalanine metabolism (*q* = 5.458 × 10^−2^), tyrosine metabolism (*q* = 3.642 × 10^−2^), and glycine, serine, and threonine metabolism (*q* = 5.428 × 10^−2^) pathways, respectively (Supplementary Figure S4A). In young fruits, 22, 8, and 55 DEGs were significantly enriched in the spliceosome (*q* = 2.931 × 10^−3^), basal transcription factors (*q* = 5.471 × 10^−2^), and RNA polymerase (*q* = 5.504 × 10^−1^) protein biosynthesis pathways, respectively (Supplementary Figure S4A,B). In both tender leaves and young fruits, 13 and 7 DEGs, respectively, were enriched in the DNA replication pathway (Supplementary Figure S4A,B). Notably, both protein biosynthesis and DNA replication are necessary for organ expansion as key components of the basic cellular processes responsible for cell division.

**Figure 4:**
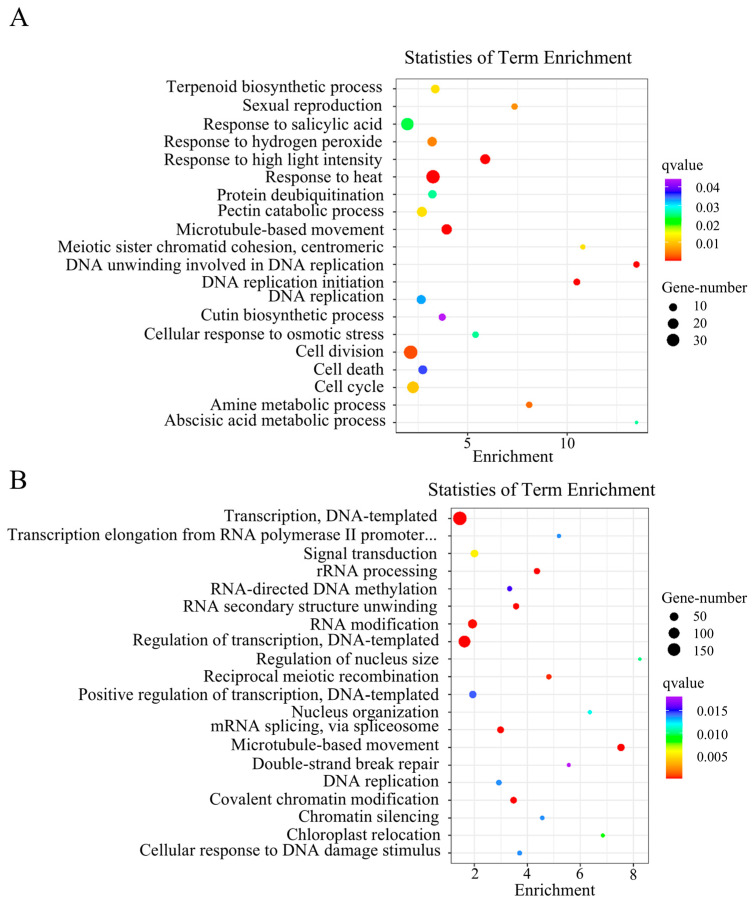
Biological process from GO enrichment analyses of DEGs in tender leaves and young fruits of mutant M25 and the WT plant Newhall navel orange. (**A**) Tender leaves; (**B**) Young fruits.

Next, GO enrichment analysis was conducted to classify the functions of all DEGs into three groups: biological processes, cellular components, and molecular functions (Supplementary Figure S5A,B; Supplementary Tables S8 and S9). Among the DEGs in both tissue types, cellular processes exhibited the greatest enrichment in biological functions (Supplementary Figure S5A,B). We further identified the 20 GO biological process terms showing the greatest enrichment in both tissue types (Figure 4A,B). DEGs in tender leaves were strongly enriched in cell division regulation processes, including cell division (*q* = 1.968 × 10^−3^), cell cycle (*q* = 1.004 × 10^−2^), DNA unwinding during replication (*q* = 4.250 × 10^−5^), and DNA replication initiation (*q* = 7.218 × 10^−5^) (Figure 4A). DEGs in young fruits were also significantly enriched in cell-division regulation processes, such as DNA replication (*q* = 1.3358 × 10^−2^) (Figure 4B), and in transcription regulation activity (Supplementary Figure S5A,B). Among biological processes, DEGs in young fruits were strongly enriched in protein biosynthesis regulation processes (Figure 4B). These findings indicate that the DEGs involved in cell division and protein biosynthesis were required for the basic cellular processes responsible for leaf and fruit size in navel orange plants.

### 3.4. Hormone-Related DEGs Regulate Organ Size in Navel Oranges Mutant M25

**Figure 5:**
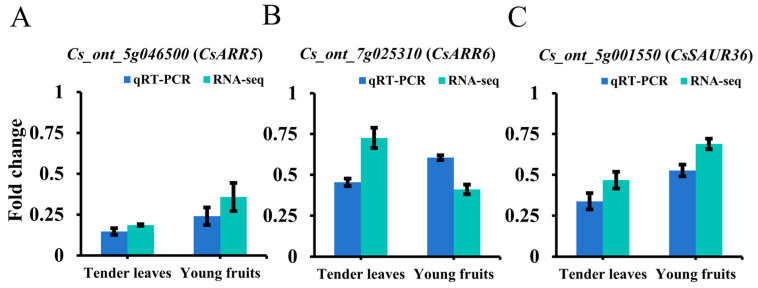
Expression analysis of 3 hormone-related genes in tender leaves and young fruits. Comparison of fold change in differential expression values determined by RNA-Seq (light blue) and qPCR (dark blue) for DEGs. (**A**) *Cs_ont_5g046500*; (**B**) *Cs_ont_7g025310*; (**C**) *Cs_ont_5g001550*.

32 and 21 enriched DEGs were enriched in plant hormone signal transduction pathways in tender leaves and young fruits, respectively (Figure 3C,D). Among these genes, 16 were associated with auxin or cytokinin (Supplementary Table S7), including *Cs_ont_5g001550* (*CsSAUR36*), which encodes an auxin-induced protein that negatively regulates plant growth by inhibiting cell expansion [35], and *Cs_ont_5g046500* (*CsARR5*) and *Cs_ont_7g025310* (*CsARR6*), which encode type-A cytokinin transcription repressors that negatively regulate plant growth by inhibiting the cytokinin signaling pathway to control cell division [36]. RNA-seq and qRT-PCR analyses of these three genes demonstrated that their expression was downregulated in the tender leaves and young fruits of the ‘M25’ mutant compared to the ‘Newhall’ navel orange plants (Figure 5A–C). These results indicate decreases (and increases) in auxin (and cytokinin) signals, which negatively (and positively) regulate organ size in ‘M25’ plants; the interplay of these effects culminates in a significantly larger organ size in this variety.

### 3.5. Cell Wall Metabolism-Related DEGs Regulate Organ Size in Navel Oranges Mutant M25

A total of 42 differentially expressed genes (DEGs) were identified as being enriched in pathways related to cell wall metabolism (*q* = 0.1569), encompassing pectin, cellulose, and lignin synthesis, as well as catabolic biological processes. Among these DEGs, 11 genes were found to be upregulated and 7 downregulated in both tender leaves and young fruits. Notably, the expression levels of exopolygalacturonase (*Cs_ont_8g029220*), which encodes a key enzyme in pectin catabolism and is involved in cell wall degradation, were 2.40- and 3.67-fold lower in the tender leaves and young fruits of ‘M25’ plants, respectively, in comparison to ‘Newhall’ plants. Additionally, the expression levels of both *Cs_ont_1g002520* and *Cs_ont_1g002540*, which encode caffeic acid 3-O-methyltransferase (COMT)—an enzyme integral to the lignin synthesis pathway—were upregulated in the tender leaves of mutant plants. Furthermore, the expression of *Cs_ont_4g020890*, which encodes cellulose synthase, was also upregulated in the young fruits of mutant plants. These alterations in the expression of DEGs associated with cell wall metabolism align with the observed phenomenon of cell enlargement.

### 3.6. Co-Expression Network Analysis

**Figure 6:**
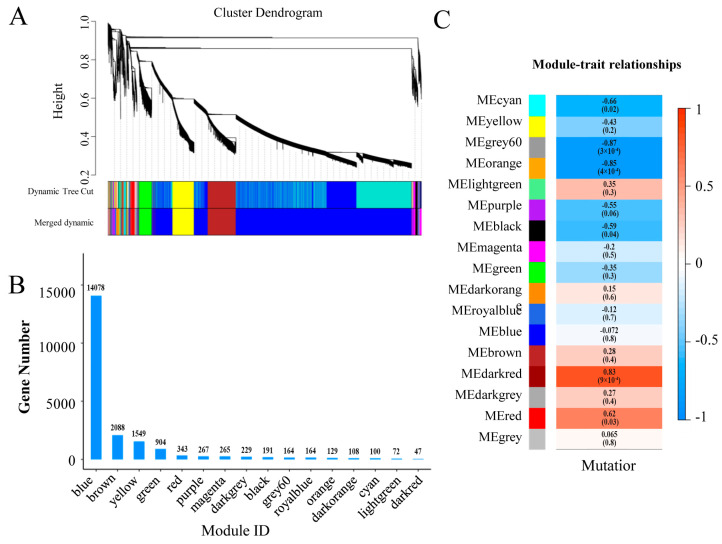
Identification of modules related to fruit ripening by gene co-expression network and module–trait relationships analysis. (**A**) Weighted gene co-expression network analysis identified 17 co-expression modules. (**B**) Number of genes in each module. (**C**) Module–trait relationships plot. The module sample correlation and corresponding *p* values are shown in parentheses. The panel on the left shows 17 modules. The color code on the right shows the module feature correlation from − 1 (blue) to 1 (red).

To identify key genes associated with the organ size of citrus plants, we constructed a weighted co-expression network utilizing the WGCNA package in R (R Core Team, Vienna, Austria). The genes were categorized into 17 merged co-expression modules (Figure 6). Most of these genes were assigned to the blue (14,078) and dark red (47) modules. Our analysis of module–trait relationships identified three modules (gray 60, orange, and dark red) that were clearly correlated with organ enlargement in ‘M25’ (Figure 6C). Within these modules, the gray 60 (R^2^ = −0.87) and orange (R^2^ = −0.85) module eigengenes were found to be negatively related to organ enlargement, whereas the dark red (R^2^ = 0.83) module eigengene was positively related to organ enlargement. Functional analysis showed that genes related to cell wall metabolism, hormones, and genetic pathways in navel orange plants were concentrated mainly in the gray and orange modules (Supplementary Figure S6A–D). Therefore, we inferred that the gray and orange modules are crucial to organ size.

**Figure 7:**
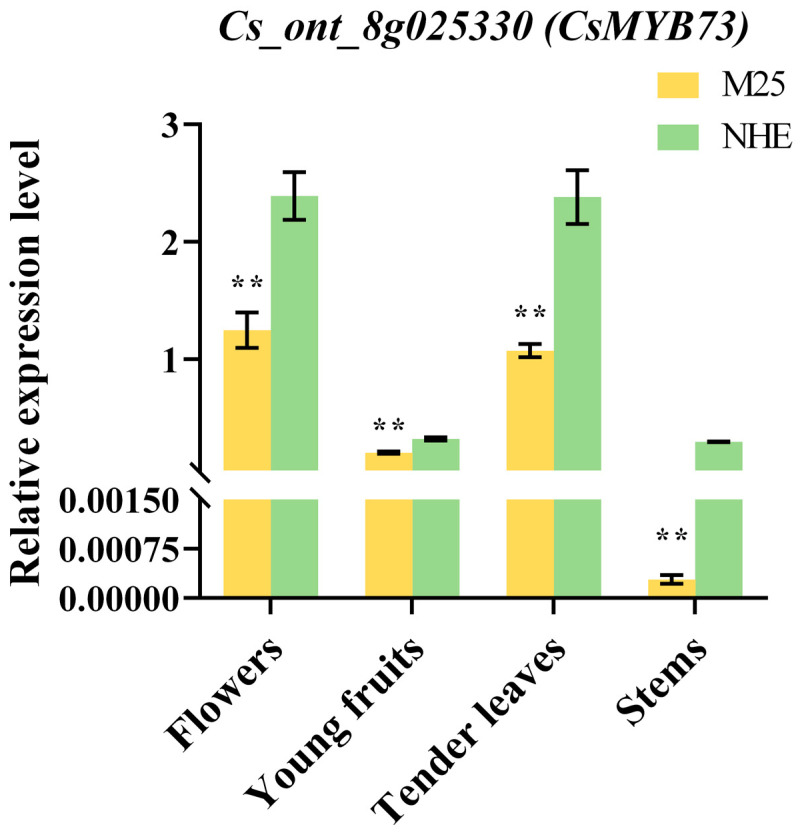
The relative expression levels of *Cs_ont_8g025330*. The error bars represent the standard error of three biological replicates. The significance analysis was performed by Student’s *t*-test; ** denotes significant at the 0.01 probability levels.

Using a threshold of >0.8 for both module feature and gene saliency values, we identified 86 hub genes within the gray 60 (51 genes) and orange (35 genes) modules. Among these, 10 genes were annotated as transcription factors, the expression of four of which (*Cs_ont_6g005380*, *Cs_ont_8g025330*, *Cs_ont_9g019400*, and *Cs_ont_9g008010*) was significantly lower in both tender leaves and young fruits in ‘M25’ than in ‘Newhall’ navel orange plants. We analyzed the gene expression levels of these four genes in tender leaves and young fruits by qRT-PCR (Figure 7, Supplementary Figure S7).

Next, we performed gene function annotation analyses of the four genes (Table 1). *Cs_ont_6g005380* encodes R2R3-MYB transcription factor 20 (MYB20). In Arabidopsis, *AtMYB20* downregulates the expression of Type 2C serine/threonine protein phosphatases (PP2Cs), which are negative regulators of abscisic acid signaling and are involved in plant hormone signaling pathways [37]. *Cs_ont_8g025330* encodes R2R3-MYB transcription factor 73 (MYB73). In Rosa × hybrida, MYB73 was found to recruit the TOPLESS co-repressor and histone deacetylase 19 (had19) to form a suppression complex that regulates MIR159 expression through modulation of histone H3 lysine 9 (H3-K9) acetylation levels at the MIR159 promoter, inhibiting accumulation of cytokinin oxidase/dehydrogenase6 (CKX6) transcripts and leading to cytokinin accumulation and a prolonged developmental cell division period [19]. *Cs_ont_9g019400* encodes the ethylene-responsive APETALA2 (AP2)-like transcription factor *NsAP2* in the water lily Nymphaea cultivar ‘Yellow Prince’; in *Arabidopsis*, *NsAP2* overexpression downregulated the expression of gibberellin oxidases GA2ox2 and GA2ox7, which negatively regulate gibberellin biosynthesis, producing more petals and taller plants [38]. *Cs_ont_9g008010* encodes ethylene-responsive transcription factor 1B (ERF1B) and *MdERF1B*, a core gene responsive to jasmonic acid and ethylene signals; the encoded protein, together with the jasmonic acid signal transduction factor *MdMYC2*, jasmonate ZIM-domain 5/10 (*MdJAZ5/10*), and ethylene insensitive-like 1 (*MdEIL1*), have been found to modulate apple tree growth and development [39]. Therefore, the four hub genes identified in this study are candidate genes potentially related to organ size.

## 4. Discussion

The leaf is a crucial organ in plant photosynthesis, providing the majority of carbohydrates necessary for plant growth and development. The size and shape of a plant leaf are significant agronomic traits correlated with photosynthetic efficiency [40]. The fruit represents a pivotal structure in angiosperms, with fleshy fruits serving as horticultural commodities that possess high nutritional value. Fruit size is a key determinant of both yield and quality in many horticultural crops that produce fleshy fruits [41]. Investigating gene functions and manipulating organ-size-related genes has enhanced fruit yield and quality in various citrus varieties. The *Citrus sinensis* variety ‘M25’, a mutant of the ‘Newhall’ navel orange, represents a novel citrus breeding germplasm characterized by enlarged organ size.

In this study, we conducted RNA-seq analysis of differentially expressed genes (DEGs) in the leaves and fruits of both navel orange varieties. We found that the fruit of the ‘M25’ mutant exhibited up to ten times more downregulated genes than upregulated genes. Previous studies based on transcriptome sequencing have similarly indicated that mutants tend to have significantly more downregulated DEGs compared to upregulated DEGs [42,43].

In recent years, numerous factors that regulate organ size have been identified, including phytohormones, transcription and elongation factors, the ubiquitin-proteasome and microRNA pathways, G-protein and receptor kinase signaling, as well as arabinogalactan and RNA-binding proteins [11]. Among these, the hormonal regulation of organ size is a key research focus, particularly the roles of auxins and cytokinins, which are crucial regulators of the dynamic balance between plant cell division and differentiation, thereby influencing organ size [36,44]. Many genes regulate organ size by modulating hormone networks; for instance, MYB73 can interact with multiple genes to inhibit the transcription of downstream genes, including cytokinin oxidase/dehydrogenase 6 (CKX6), auxin-responsive factors (ARFs), and gibberellin 2-oxidase (GA2ox1), all of which are involved in the regulation of complex hormone networks affecting plant organ size [19,45,46]. In this study, a total of 43 differentially expressed genes in ’M25’ and ’Newhall’ were enriched in hormone signal transduction pathways. Among these, 16 genes were associated with cytokinin and auxin, leading to alterations in cytokinin and auxin signaling in ’M25’ compared to the Newhall navel orange, which resulted in organ enlargement in ’M25’.

Many studies have analyzed the causes of changes in plant organ size from the perspective of cell wall metabolism. Cell wall relaxation serves as the foundation for rapid cell enlargement, with cell wall esterases and cellulose–hemicellulose networks playing crucial roles in cell wall remodeling [47]. Jin found that the soybean GRF-interacting factor gene (*GmGIF5*) promotes cell expansion by negatively regulating cell wall modification. Overexpression of *GmGIF5* resulted in larger leaves, taller plants, wider stems, and larger seeds by increasing cell size rather than cell number [48]. Conversely, Wang’s research on rice revealed that the knotted Arabidopsis thaliana 7 protein gene (KNAT7) coordinates cell wall relaxation and reinforcement by inhibiting the expression of cellulose synthase genes (CESAs) and Expansins. Mutants of KNAT7 exhibit cell wall thickening and abnormal cell expansion, leading to a reduction in cell numbers [49]. This indicates that cell wall metabolic pathways can simultaneously influence both cell division and expansion in plants, regulating cell size and cell number through complex regulatory networks and ultimately controlling the growth and development of plant organs.

Cytological observations of paraffin sections have revealed significant differences in the cell area of leaf spongy tissue and palisade tissue between various plant varieties [50,51]. In most plants, organ enlargement results from the synergistic effects of cell division and cell expansion, particularly under the influence of hormone-regulated genes [45,51,52,53]. In this study, the leaves of ‘M25’ plants were larger than those of ‘Newhall’ plants, and we observed an increase in the cell area of both spongy and palisade tissues in ‘M25’ plants, while no difference was noted in the epidermal cell area. Plants with identical numbers and areas of epidermal cells cannot sustain a larger leaf area. Consequently, the greater number of epidermal cells in ‘M25’ plants contributed to the formation of larger organs compared to ‘Newhall’ plants, with cell division and expansion further synergistically enhancing these organs.

MYB transcription factors represent one of the most widespread families of transcription factors in plants, participating in various physiological activities through their interaction with MYB cis-elements in the promoters of target genes. They are key components in regulatory networks that govern plant development, metabolism, and responses to both biotic and abiotic stresses [54,55]. Numerous studies have established a relationship between MYB transcription factors and fruit ripening, fruit size, carotene and chlorophyll contents, anthocyanin metabolism, and lignin biosynthesis in citrus varieties [20,56,57,58,59]. Our WGCNA identified the MYB73 gene (*Cs_ont_8g025330*) as a potential key regulator of organ size. Research by Jing et al. [19] and Lorenzo et al. [60] has demonstrated that MYB73 regulates several hormones; for instance, during rose petal development, MYB73 forms an inhibitory complex with TPL/HDA19, which reduces the expression of MIR159, leading to an accumulation of cytokinin oxidase 6. This process results in cytokinin clearance and subsequent cell division arrest, ultimately reducing flower size [19]. In *Arabidopsis*, MYB73 functions as an auxin response transcription factor that promotes auxin signaling, thereby influencing organ growth [45]. These findings indicate that downregulation of MYB73 expression may enhance cytokinin and auxin signaling, positively regulating cell division and expansion. In soybeans, MYB73 decreases gibberellin 2-dioxygenase content by inhibiting agophosphoryl diesterphosphodiesterase activity, which promotes gibberellin accumulation [60]. Furthermore, MYB transcription factors play a critical role in the biosynthesis of secondary metabolites in plants, including anthocyanins, flavonols, and lignin [54]. WGCNA of wild grape (*Vitis adenoclada*) fruit revealed a strong correlation between high COMT expression and MYB73 [61]. This finding aligns with the current results, which indicate that the expression levels of *Cs_ont_1g002520* and *Cs_ont_1g002540*—key COMT genes involved in lignin synthesis and cell wall metabolism—were upregulated in the ‘M25’ mutant. Consequently, the MYB73 gene (*Cs_ont_8g025330*) may function as a crucial regulator of citrus organ size by coordinating auxin, gibberellin, and cytokinin signaling networks, as well as modulating the roles of COMT in lignin synthesis and cell wall metabolism.

## 5. Conclusions

Through a comparative analysis of phenotype, cytomorphometric characteristics, and chromosome ploidy of the ‘Newhall’ navel orange and its enlarged organ size mutant ‘M25’, we found that the enhanced growth of organs in ‘M25’ was influenced by enlarged cell size rather than changes in ploidy. RNA-seq and WGCNA analysis indicated that cell division, DNA replication and protein biosynthesis, plant hormone signal transduction, and cell wall metabolism were the critical pathways related to organ size. Additionally, four hub genes encoding transcription factors that regulate organ size were identified. Among these, *Cs_ont_8g025330* was inferred to be the key gene that regulates downstream genes to coordinate the auxin and cytokinin signaling networks. Together, our findings provide insights into citrus organ size and establish a foundation for breeding citrus cultivars with modest fruit sizes and higher yields.

## Figures and Tables

**Table 1:** Gene expression and function of important transcription factors in gray60 and orange modules.

Gene ID	Gene	Gene Function	Leaf		Fruit	
			M25	NHE	M25	NHE
*Cs_ont_6g005380*	*CsMYB20*	Transcription factor MYB20	0.802 ± 0.153 **	2.308 ± 0.259	0.872 ± 0.509	1.730 ± 0.191
*Cs_ont_8g025330*	*CsMYB73*	Transcription factor MYB73	1.741 ± 0.322 *	3.219 ± 0.157	2.215 ± 0.689	3.100 ± 0.613
*Cs_ont_9g008010*	*CsERF1B*	Ethylene-responsive transcription factor	0.194 ± 0.018 **	1.017 ± 0.500	0.0177 ± 0.015 *	0.608 ± 0.352
*Cs_ont_9g019400*	*CsAP2*	AP2-like ethylene-responsive transcription factor	8.395 ± 0.554 **	14.548 ± 0.431	7.676 ± 3.505	11.539 ± 1.497

We examined the expression of *Cs_ont_8g025330* (*CsMYB73*) in ‘M25’ and ‘Newhall’ navel orange plants. Our findings revealed that the expression of CsMYB73 was 0.85- and 0.40-fold lower in the tender leaves and young fruits of ‘M25’, respectively. The relative expression levels of *CsMYB73* in the flowers and stems of ‘M25’ and ‘Newhall’ were assessed using qRT-PCR. The results indicate that *CsMYB73* was significantly downregulated in the flowers and stems of ‘M25’ plants compared to those of ‘Newhall’, consistent with our findings for tender leaves and young fruits (Figure 7). Therefore, *CsMYB73* is a key gene involved in increased organ size and may play an important role in downstream gene regulation; * denotes significant at the 0.05 probability levels, ** denotes significant at the 0.01 probability levels.

## Data Availability

RNA-seq data of this study have been uploaded to the National Biotechnology Information Center (NCBI) SRA database with the primary accession code PRJNA1173850.

## Supplementary Materials

The following supporting information can be downloaded at: https://www.mdpi.com/article/10.3390/genes16030259/s1, Figure S1: Phenotypic analysis of stem and fruit of M25 and Newhall navel orange. (A) Diameter of the mature stem; (B-C) single fruit weight and transverse diameter of fruit at days after flowering 180, 200, and 220. ⁎⁎ denotes significant at the 0.01 probability levels; Figure S2: Cell size in paraffin sections of mature leaves between M25 and Newhall navel orange. ⁎ and ⁎⁎ denote significant differences at the 0.05 and 0.01 probability levels, respectively; Figure S3: Differentially expressed volcano map. (A) Tender leaves; (B) Young fruits; Figure S4: Scatterplot of KEGG pathway enrichment of DEGs. (A) Tender leaves; (B) Young fruits; Figure S5: GO-annotated classification statistical map of DEGs. (A) Tender leaves; (B) Young fruits; Figure S6: Enrichment analyses of genes in grey60 and orange module. (A) KEGG enrichment analysis of genes in the grey60 module; (B) Biological_process (GO) enrichment analyses for genes in the grey60 module; (C) KEGG enrichment analysis of genes in the orange module; (D) Biological_process (GO) enrichment analyses for genes in the orange module; Figure S7: Three DEGs’ relative expression levels as determined by qRT-PCR. (A) Cs_ont_9g008010; (B) Cs_ont_6g005380; (C) Cs_ont_9g019400. The error bars represent the standard error of three biological replicates. ⁎⁎ denote significant at the 0.01 probability levels; Table S1: General statistics of sequencing data; Table S2: Primers used for qRT-PCR in this study; Table S3: Chl contents of M25 and Newhall navel orange; Table S4: Identification of differentially expressed genes related to leaf size between M25 and Newhall navel orange; Table S5: Identification of differentially expressed genes related to fruit size between M25 and Newhall navel orange; Table S6 KEGG enrichment analysis of all DEGs in tender leaves of M25 navel orange; Table S7 KEGG enrichment analysis of all DEGs in young fruits of M25 navel orange; Table S8: GO enrichment analysis of DEGs in tender leaves of M25 navel orange; Table S9: GO enrichment analysis of DEGs in young fruits of M25 navel orange; Table S10: Important differentially expressed genes of plant hormone signal transduction pathways related to organ size in tender leaves and young fruits M25 navel orange; Table S11: Important differentially expressed genes of cell wall metabolism pathways related to organ size in tender leaves and young fruits M25 navel orange; Table S12 Gene expression and functional annotation of four important transcription factors.
