# Supplementary material for: Identification of Key Pathways and Candidate Genes Controlling Organ Size Through Transcriptome and Weighted Gene Co-Expression Network Analyses in Navel Orange Plants (Citrus sinensis)
Source: Genes (Basel). 2025 Feb 23;16(3):259. doi: 10.3390/genes16030259 (PMC11942113; doi:10.3390/genes16030259)
Supplement: Supplementary file 1 [file genes-16-00259-s001.zip › Supplemental Figure.pdf]

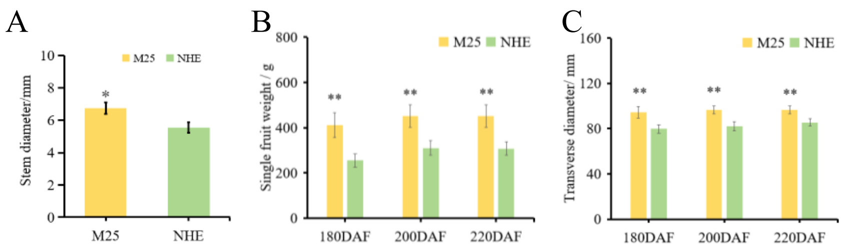

Supplemental Figure S1 Phenotypic analysis of stem and fruit of M25 and Newhall navel orange. (A) Diameter of the mature stem; (B-C) Single fruit weight and transverse diameter of fruit at days after flowering 180, 200 and 220. ∗∗ denote significant at the 0.01 probability levels.

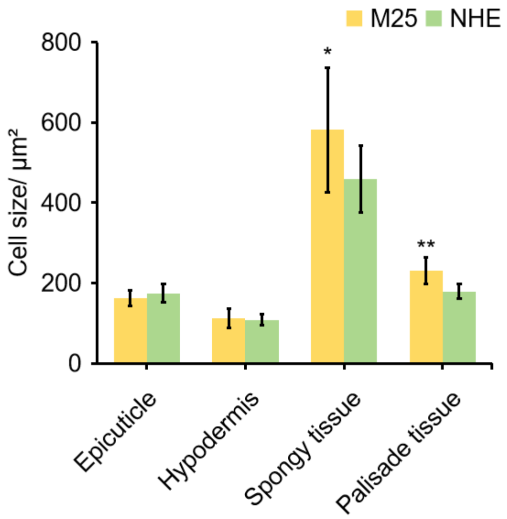

Supplemental Figure S2 Cell size in paraffin sections of mature leaves between M25 and Newhall navel orange. * and ** denote significant difference at the 0.05 and 0.01 probability levels, respectively.

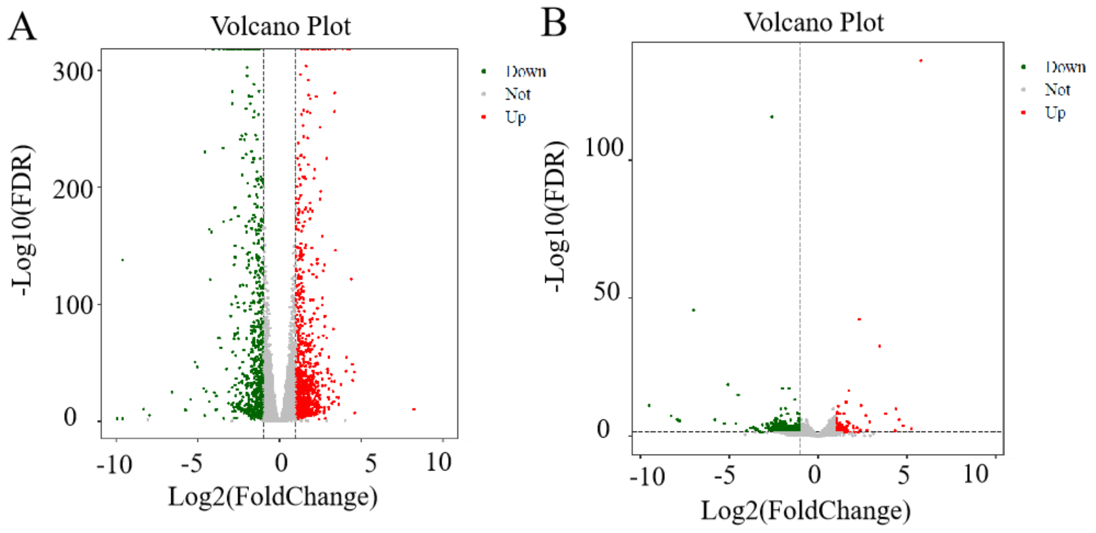

Supplemental Figure S3 Differentially expressed volcano map. (A) Tender leaves; (B) Young fruits.

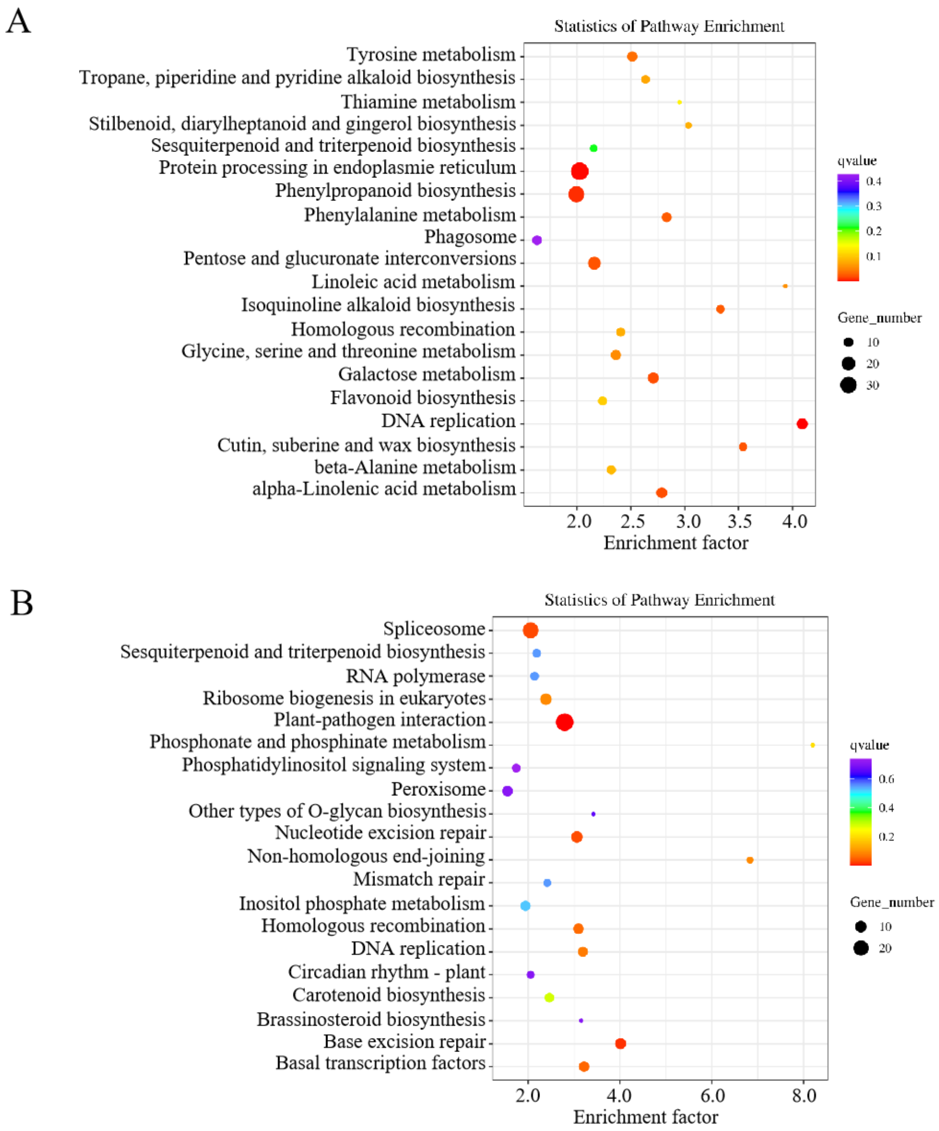

Supplemental Figure S4 Scatterplot of KEGG pathway enrichment of DEGs. (A) Tender leaves; (B) Young fruits.

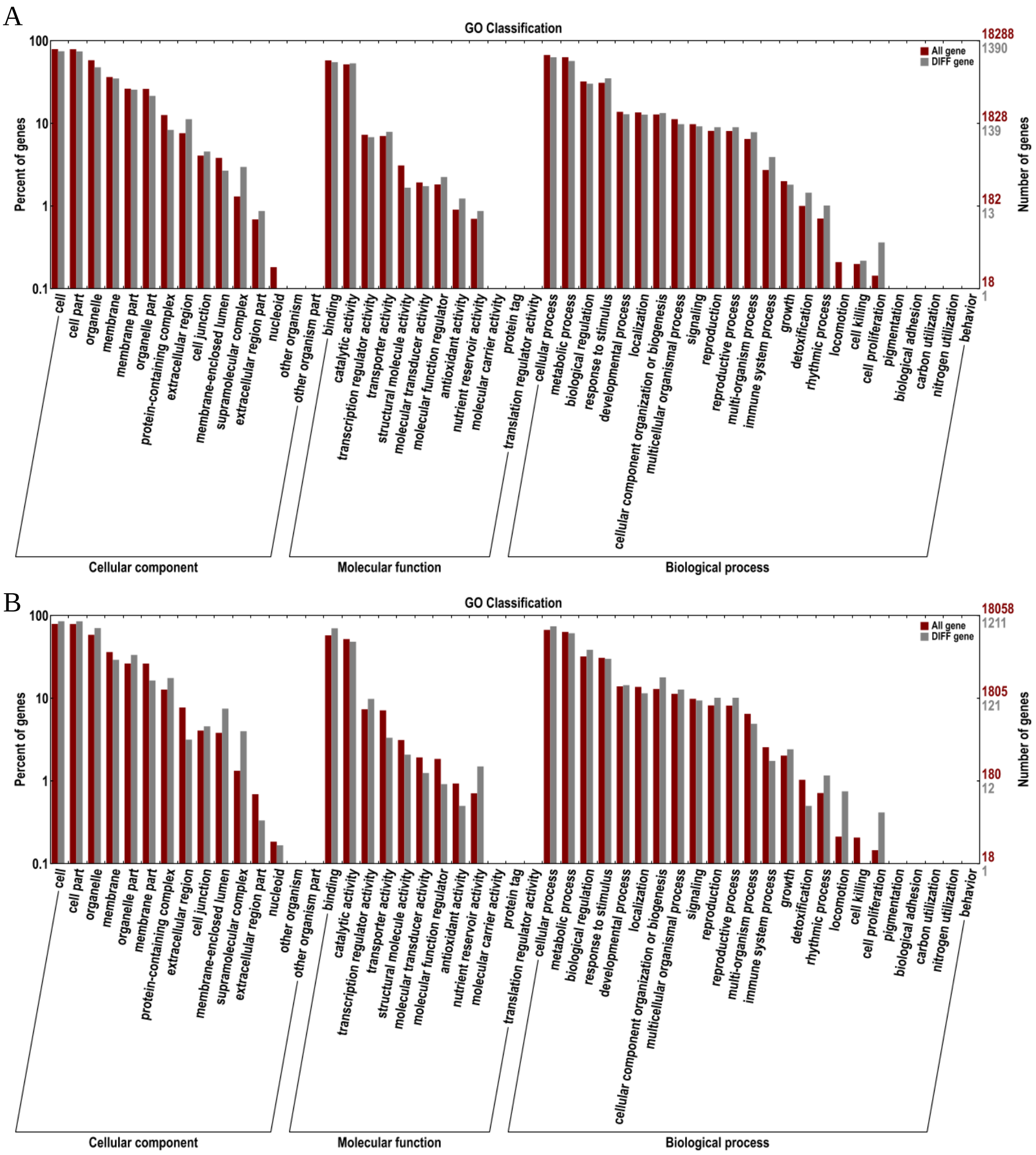

Supplemental Figure S5 GO annotated classification statistical map of DEGs. (A) Tender leaves; (B) Young fruits.

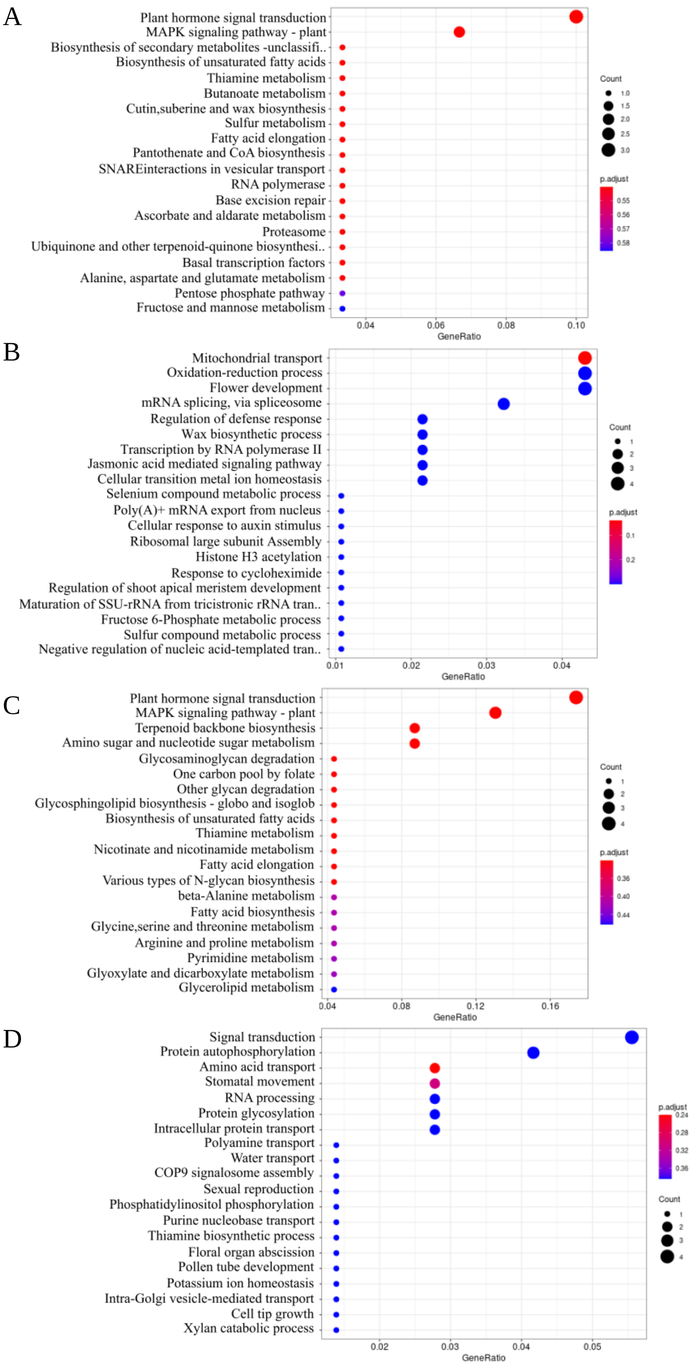

Supplemental Figure S6 Enrichment analyses of genes in grey60 and orange module. (A) KEGG enrichment analysis of genes in grey60 module; (B) Biological_process (GO) enrichment analyses for genes in grey60 module; (C) KEGG enrichment analysis of genes in orange module; (D) Biological_process (GO) enrichment analyses for genes in orange module.

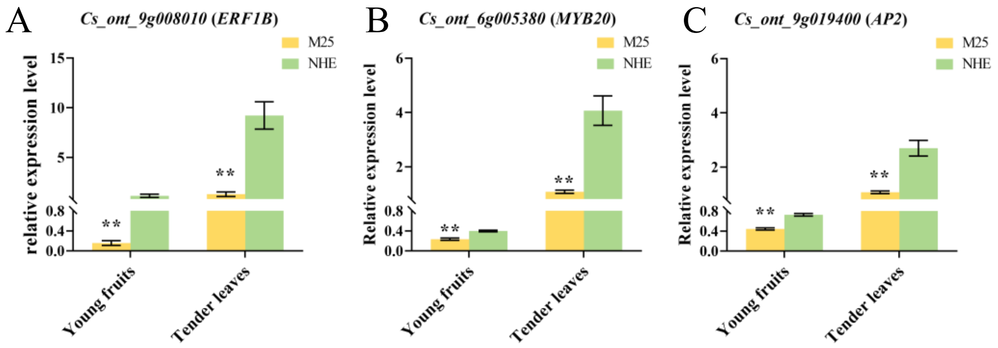

Supplemental Figure S7 Three DEGs' relative expression levels as determined by qRT-PCR. (A) *Cs_ont_9g008010*; (B) *Cs_ont_6g005380*; (C) *Cs_ont_9g019400*. The error bars represent the standard error of three biological replicates. ∗∗ denote significant at the 0.01 probability levels.
